# Acceptability of evidence-based neonatal care practices in rural Uganda – implications for programming

**DOI:** 10.1186/1471-2393-8-21

**Published:** 2008-06-21

**Authors:** Peter Waiswa, Margaret Kemigisa, Juliet Kiguli, Sarah Naikoba, George W Pariyo, Stefan Peterson

**Affiliations:** 1 Makerere University School of Public Health, Kampala, Uganda; 2 Iganga District Health Department, Iganga, Uganda; 3 International Health, Dept of Public Health Sciences (IHCAR), Karolinska Institutet, Sweden; 4 Saving Newborn Lives, Save the Children USA, Uganda field office, Uganda; 5 International Maternal and Child Health, Dept of Women's and Children's Health, Uppsala University, Sweden

## Abstract

**Background:**

Although evidence-based interventions to reach the Millennium Development Goals for Maternal and Neonatal mortality reduction exist, they have not yet been operationalised and scaled up in Sub-Saharan African cultural and health systems. A key concern is whether these internationally recommended practices are acceptable and will be demanded by the target community. We explored the acceptability of these interventions in two rural districts of Uganda.

**Methods:**

We conducted 10 focus group discussions consisting of mothers, fathers, grand parents and child minders (older children who take care of other children). We also did 10 key informant interviews with health workers and traditional birth attendants.

**Results:**

Most maternal and newborn recommended practices are acceptable to both the community and to health service providers. However, health system and community barriers were prevalent and will need to be overcome for better neonatal outcomes. Pregnant women did not comprehend the importance of attending antenatal care early or more than once unless they felt ill. Women prefer to deliver in health facilities but most do not do so because they cannot afford the cost of drugs and supplies which are demanded in a situation of poverty and limited male support. Postnatal care is non-existent. For the newborn, delayed bathing and putting nothing on the umbilical cord were neither acceptable to parents nor to health providers, requiring negotiation of alternative practices.

**Conclusion:**

The recommended maternal-newborn practices are generally acceptable to the community and health service providers, but often are not practiced due to health systems and community barriers. Communities associate the need for antenatal care attendance with feeling ill, and postnatal care is non-existent in this region. Health promotion programs to improve newborn care must prioritize postnatal care, and take into account the local socio-cultural situation and health systems barriers including the financial burden. Male involvement and promotion of waiting shelters at selected health units should be considered in order to increase access to supervised deliveries. Scale-up of the evidence based practices for maternal-neonatal health in Sub-Saharan Africa should follow rapid appraisal and adaptation of intervention packages to address the local health system and socio-cultural situation.

## Background

The Millennium Development Goal (MDG) 4 – to reduce under-five mortality by two thirds, and MDG 5 – to reduce maternal mortality by three-quarters between 1990 and 2015 [1] are related. Maternal interventions also benefit children, especially in relation to newborn survival [2]. Globally, it is estimated that 4·0 million neonatal deaths occur every year [3,4], 25% of whom are from Africa [4,5]. In addition, over 515,000 women die from pregnancy-related complications each year, about half in Africa [6]. Most of these maternal and newborn deaths are caused by preventable causes [4,7].

Two recent *Lancet series*, one on Newborn health [2] and another on Maternal health [6] have proposed key evidence-based interventions and packages which, if implemented to scale, could greatly contribute to saving maternal and newborn lives in low income countries. The interventions emphasize strengthening the continuum of maternal, newborn and child care including antenatal care (ANC), intrapartum care and postnatal care for the mother and the newborn [8-10]. However, it is not clear how feasible and locally acceptable these recommended practices are, or which delivery strategies to use in Sub-Saharan Africa (SSA). Most recommendations are based on studies conducted in Asia and South America [11-13] and so far almost none from SSA where the epidemiology, culture and health systems are different.

In Uganda, the neonatal and maternal mortality rates remain high at 29/1000 and 435/100,000 live births respectively [14]. Only 42% of pregnant women get a supervised delivery (defined here as a delivery by a trained health professional – that is, a doctor, nurse, midwife, medical assistant or clinical officer); and emergency obstetric care (EmOC) met need is only 14% [15,16]. Birth preparedness (BP) – a package that promotes planning for delivery and the coming baby [17], is almost non-existent. New policies prioritizing delivery of integrated maternal-newborn programs including use of community strategies have been developed [18,19], but these have not yet been operationalized.

In order to inform the design of a community-based maternal and neonatal health care intervention, we conducted formative research to explore the acceptability and barriers to the recommended evidence-based practices and to home-visiting by a community volunteer to improve practices along the continuum of maternal and newborn care for better neonatal outcomes.

## Methods

This study was undertaken in two rural districts of Iganga and Mayuge during December 2006 and January 2007 in Busoga region, Eastern Uganda. The Busoga region has seven districts and about 3 million people, or 10% of Uganda's population. Ten focus group discussions (FGDs) were conducted as follows: two with younger mothers less than 30 years; four with older mothers more than 30 years or having grandchildren; two with fathers and another two with child minders (older children who take care of other children) of up to 13 years of age. Selection of young mothers and fathers was limited to those having children less than six months of age in order to ensure that responses reflect recent/current practices. In addition, we also conducted key-informant interviews (KIs) with six health workers and four traditional birth attendants (TBAs).

Villages were selected for interviews from both near and far from the hospital to represent the rural-urban divide. Using guidelines from the research team, community leaders identified participants for the FGDs, and district leaders of health services identified health workers and TBAs for the KIs. Pre-tested checklists guided discussions about the acceptability and barriers to adapting practices within the continuum of care approach [9,10,20] with special focus on ANC, intra-partum care, and postnatal care for the mother and the baby, and to home visits by a volunteer to promote improved care during pregnancy, delivery and in the postnatal period. Participants were asked to present their own experiences and actions, or otherwise to describe general attitudes.

Interviews with health workers were conducted in English, tape-recorded, transcribed and compiled with field notes. Interviews with TBAs and all the FGDs were conducted in the local language, *Lusoga*, tape-recorded and transcribed by the moderators. Two *Lusoga *speakers independently translated interviews into English, leaving all local terminologies in *Lusoga *to keep informative words intact.

Analysis of the in-depth interviews (IDIs) and FGDs used latent thematic content analysis. Transcripts were first read several times to get an overall picture and then meaningful units were coded, condensed and categorized into broad themes [21]. Barriers to care seeking were characterized according to the three delays model which includes delays in deciding to seek care, delay in reaching the health facility, and delay in receiving care once at the health facility [22,23].

The study tools were developed in consultation with national policy makers who included the Iganga and Mayuge districts, the Ministry of Health, the World Health Organization (WHO), UNICEF, and Saving Newborn Lives (SNL) Uganda field offices. An experienced social scientist and a medical doctor trained and supervised the research assistants during pilot testing and field work. All moderators were experienced and their minimum education was to diploma level. Verbal informed consent was sought and obtained from all participants. The study was approved by the Makerere University School of Public Health Institutional Review Board and the Uganda National Council for Science and Technology.

## Results

### ANC Practices

Few women reported attending ANC four times during pregnancy. The reasons given for this included knowledge barriers and service delivery gaps; cultural, traditional beliefs and practices; and financial constraints. These are summarized in table 1 according to the three delays model

**Table 1:** Barriers to birth preparedness and antenatal-care attendance according to the three delays model [22,23], in Busoga, Eastern Uganda

**DELAY**	**BARRIER**
**DELAY 1: DELAY TO SEEK CARE**	

**Knowledge Barriers**	- ANC misconstrued as provision of medicine for sick pregnant women - Limited community knowledge on: importance of attending ANC four times; importance of ANC to mother and unborn baby; cause and care for danger signs
**Culture and traditional beliefs and practices**	- Deep rooted beliefs in herbs as part of pregnancy care - Decision making as a male prerogative (seeking and choice of care) - Conflicts related to polygamy: men making preferential treatment among wives - Mother-in-laws making decisions for daughter-in-laws - Influence from older mothers - Too much burden of work on women - The fear of preparing for the unborn whose viability is considered uncertain

**DELAY 2: DELAY TO ARRIVE AT A HEALTH UNIT**	

**Financial Constraints**	- Lack of money for transport and hospital related costs (including under the table payments) - Women's reliance on male partners for funds and men unable to raise and sometimes unwilling to give the funds - Health facility requirements for BP being too costly for families
**Peer influence on choice of care**	- Women relying on fellow women for advise on ANC attendance

**DELAY 3: DELAY TO GET CARE ONCE AT A HEALTH UNIT**	

**Service delivery gaps**	- Emphasis on ANC card by health workers as a pre-condition to skilled care - Lack of skilled staff - Poor attitude and communication skills of health workers e.g. rudeness and ignoring clients - Health workers do not actively encourage couples to choose skilled providers for delivery

In Busoga, ANC attendance is called "*okunhwa obulezi"*, literally translated *"drinking medicine"*. Thus, the community associates ANC with being "ill". Consequently, only women who feel *"ill" *do attend ANC and when they do so they expect to get a lot of medication. For instance, intermittent presumptive treatment (IPT) of malaria with fansidar is one of the key interventions to reduce malaria in pregnancy and improve maternal and fetal outcomes. However, when women get only three tablets of fansidar (the appropriate treatment), they get frustrated, as was reported in one FGD:

*"Yes, I was given three fansidars and they are at home. I came back quarrelling. I went for ANC for assistance but by giving me only three tablets, how were they helping? Three tablets only! Yet I explained my condition in detail"*. (FGD, Young Mothers).

TBAs are perceived as effective care-givers since they provide herbal medicine, and as more mature providers with 'better' personalized care compared to health workers. Furthermore, TBAs are easily accessible to the community day and night. In addition, it was believed that herbs from TBAs give magical effects which modern medicine may not be able to provide.

*"Yes, she (TBA) delivers and also changes the position of the baby if it is not laying right. She can also change the sex of the baby if you want. For instance if you have been giving birth to only boys and you want a girl, she can change the sex for you so that you deliver a girl" *(FGD, older mothers).

Herbs are believed "to make pelvic bones flexible at the time of delivery" so as to help women avoid the risk of caesarean birth, which they associate with delivery from hospitals. Other cases where herbs are used included malaria and syphilis treatment.

Financial constraints are a critical barrier for women who know the importance of four ANC visits, and some women attend ANC only once to get a card. As one young woman in a focus group put it:

"When I conceive and I am four months pregnant, I start going to hospital. However, what makes it difficult for me is the transport problem. Altogether, it is Shs. 4,000 to and from the hospital and also when you get to the hospital, the health workers ask for some money..... This problem causes most of us to go for ANC in the late stages of pregnancy just to get a book".(FGD, young mothers).

Despite these challenges, respondents said that attending ANC four times during pregnancy is acceptable if the barriers are removed.

### Birth Preparedness

Birth preparedness (BP) promotes active preparation and decision-making for births, including pregnancy/postpartum periods, by pregnant women and their families. Couples are sensitized about BP in ANC but implementation is poor due to limited knowledge, financial ability, and strong traditional beliefs. Participants revealed to us that men are a key barrier to BP since women rely on them for decisions and support including finances for pregnancy related care.

*"The mother tries to explain to him all she requires to go with like the attendance book, transport and money to buy something to eat as she waits to be served. The husband will tell her he has no money. What the woman will do is to sit home and not go to hospital till the time of delivery. Remember this woman has swollen legs; she is anaemic or is bleeding! But she is sitting at home" *(Health worker)

**Figure 1:**
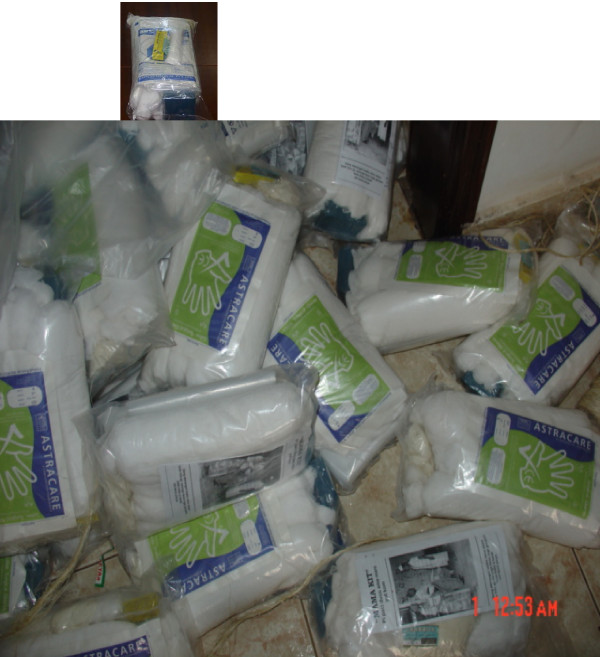
A picture of the *maama *kit – a birth-preparedness package promoted in Uganda.

In an effort to improve BP, the Uganda Ministry of Health tries to provide *maama kits *for pregnant women. However, these are often out of stock in health units, thus requiring couples to buy them. Figure 1 shows a picture of the *maama *kit – a birth-preparedness package promoted in Uganda.

**Figure 2:**
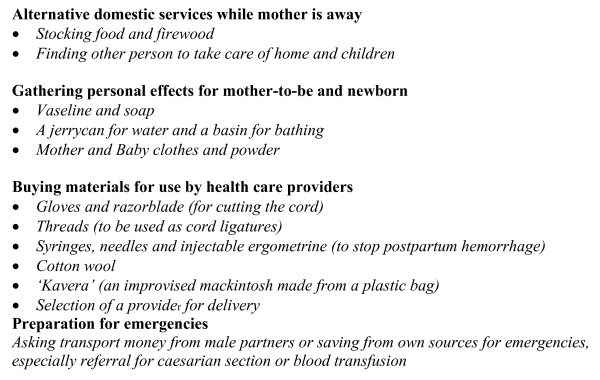
Current birth preparedness practices cited in Busoga, Eastern Uganda.

We group current birth preparedness practices in this region into four categories as shown in Figure 2:

### Care seeking for danger signs

Participants were able to identify some maternal danger signs including high fever (*omusudha*), severe pre-eclampsia (*amakiro*), hemorrhage (*okuva omusayi*), abdominal pain (*okulumwa munda*), and swelling of feet (*ebigere okuzimba*). *Amakiro *or severe pre-eclampsia was attributed to a partner being promiscuous. Other problems which respondents identified as danger signs but not necessarily medically recognized as such included syphilis (*kabotongo*) and vomiting (*okusesema*). We found that in the community, danger signs are usually first treated with herbs, and they only seek medical care when the condition becomes worse.

### Skilled attendant at delivery

Participants told us that they prefer to deliver in health units, but they usually do not do so due to the many barriers such as the expensive *maama kit*s required in health units for delivery, labour starting at night in absence of transportation and inaccessible health units that are often closed at night. Other barriers cited were health workers' rudeness, corrupt tendencies and absenteeism from work.

Given these barriers in accessing the formal health system, the possibility of getting services on credit from TBAs to offset delivery expenses was considered an easier option.

*"Usually, the labour pains begin at night. For all my four children, the labour pains began late at night at around 2.00 o'clock. But still, I would get up immediately and look for a health worker where to go. Sometimes the pains start when I don't even have any money, so I request my neighbour to help me with a bicycle and they take me. Now in my preparation bag for delivery, I usually put a good gomesi [dress], so after delivery, if I am not able to pay, I leave that gomesi behind with her [TBA] until I can pay" *(FGD, older mothers).

### Postpartum care for the mother and baby

Respondents were not aware of the need for postnatal health care attendance except for immunization of children. In one of the FGDs, postnatal care as part of maternal and newborn care was considered something new:

*"...and we just neglect making those postnatal visits yet there are complications we develop after delivery, because we usually fall ill some times after giving birth" *(FGD, young mothers).

The newborn care practices which were deemed acceptable to the community included: maintenance of warmth and cleanliness, exclusive breast feeding and skin-to-skin contact at birth. However, the recommended evidence based practices of delayed bathing and putting nothing on the umbilical cord met strong objections from both the community and health workers, as babies are believed to be born 'dirty', with a 'bad smell', and as such needed to bathe so as not to feel "uncomfortable". Early bathing of newborns is a common practice among health workers, TBAs and the community.

*"My babies are usually born dirty, so it is a must for me to bathe the baby immediately I am discharged on that same day of giving birth" *(FGD, Older mothers).

However, women were agreeable to the suggestion to wipe the baby with a wet warm clean cloth instead of early bathing.

For both facility and home births, the umbilical cord is usually cut with some form of 'sterile' equipment, usually a new razorblade. Cord-care practices included application of substances such as baby powder, spirit, herbs, soapy water, and salty water. These substances are believed to help the cord to dry/heal faster to enable women get back to their routine chores early. The fast healing of the cord is also believed to stop the pains that women feel following birth of a newborn baby. We also found that a baby whose cord has not fallen off is traditionally kept inside the house.

Interestingly, most newborn danger signs were well known to all participants including child-minders. When they were asked which signs show that a baby's health may be in danger, participants mentioned inability to suckle, weak or abnormal cry, lethargy, vomiting, fever, difficult breathing and convulsions. Only jaundice and umbilical redness were not recognized as newborn danger signs. However, due to the already outlined barriers, care seeking for newborn danger signs was reported to be poor. Table 2 outlines the perceived acceptability of recommended newborn care practices and their associated barriers.

**Table 2:** Acceptability and barriers to evidence based newborn care practices as stated in Focus Group discussions in Busoga, Eastern Uganda.

**Recommended Newborn practices**	**Perceived acceptability**	**Barriers to the practice**	**Remarks**
Cutting the cord with a clean instrument	+++++	- Difficult in home deliveries - Poor birth preparedness	- Usually a new razorblade is used
Maintenance of warmth	+++++	- Lack of money for baby clothes	- Some mothers improvise with their own used clothes
Delayed bathing	+	- Belief that babies are born "dirty" and with blood - Belief that babies who are not bathed "smell" bad - Mothers prefer that visitors find babies clean - Health care workers promote early bathing - Belief that early bathing prevents infections	- Babies bathed on day of delivery and thereafter an average of three times a day - Wiping the baby with a wet cloth could be an alternative
Maintenance of cleanliness	+++++	- Lack of money - Practice of hand washing not common	- Some wash but without soap
Exclusive breast feeding	+++++	- Colostrum not perceived to be good for the baby - Perceived lack of milk in the breast at birth	- Babies given water and/or glucose at birth
Skilled care seeking for danger signs	+++	- Lack of money - Married women often have to seek permission from husbands on choice of care - Lack of access to well equipped facilities - Health workers lack skills in managing sick newborns	- Child minders report danger signs to mothers - Both mothers and fathers appreciate importance of seeking immediate care from skilled providers
Practicing clean cord care	++	- Belief that substances applied to cord help it heal fast - Cultural practice of seclusion till cord falls off - Health workers encourage do not encourage dry care	- Mothers are under pressure for cord to heal so that they can return to routine chores - Health workers encourage application of salty water and spirit
Postnatal check up for newborns at health unit in the first week	+++++	- Practice not promoted by health system - Lack of money for transport - Lack of transport facilities	- Health workers think this will add to their already big work load
Postnatal check up for newborns at home by a volunteer in the first week	+++++	- Identifying new deliveries difficult - Motivating volunteers	- Community expects drugs at home

Key: The number of crosses reflects the degree of acceptability of the practice

### Acceptability of home visits by a community volunteer

All participants welcomed the proposed intervention to improve care of mothers and newborn babies through home visits to households by community volunteers during pregnancy and in the first week after delivery.

*"It is a very good suggestion, we accept, we are overjoyed, eh"*, (FGD for young mothers. They all clapped because they were happy with the idea).

*"We are very happy about it because trained people would be paying us a visit to establish whether mother and baby are fine or not" *(FGD, Older mothers).

Respondents mentioned the following as the desired qualities of community volunteers who would visit homes: being a woman who is a permanent resident in the area; literate and well behaved and respected in the community; ability to walk long distances; good tempered; knowledgeable; punctual; a good listener; confident; experienced in parenting; properly married, and a kind hearted person.

## Discussion

Recent reviews have suggested that promotion of certain key practices in health facilities and at community level could greatly reduce maternal and neonatal mortality in developing countries [2,8]. Consequently, experts have advocated for rapid scale-up of these evidence based interventions in developing countries. In this cultural group, we found that most of the evidence based practices which have been proposed are acceptable to both the community and to health service providers. However, practicing them is hindered by both health system and community barriers. Therefore, there is need for both health systems upgrade [24] and a strong, locally appropriate, behavior-change communication strategy as essential parts of any community-based intervention [25] to improve maternal/neonatal practices.

### Improving ANC utilization

Attending ANC four times is one of the major WHO recommended interventions for pregnant women, as it is associated with improved maternal and neonatal outcomes [26,27]. However, we found this to be rare in practice, but was said to be acceptable to the community under study if access barriers were minimized. We believe that in this setting an intervention will need to discuss at least three issues on ANC: the perception of ANC as being for the *ill *pregnant women; the role of TBAs; and attendance 'to get an ANC card'. In Uganda, it is a common reason for women to attend ANC to get a card as nurses and midwives usually compliment women who present these cards at delivery as good mothers who have been in contact with the health facility during ANC [28].

The low attendance of ANC four times during pregnancy may be partly explained by the local perception among this *Basoga *ethnic group of ANC as "*okunhwa obulezi"*, literally translated "drinking medicine". As such, the community perceives attending ANC early or more than once to be for pregnant women who are 'unwell' and only the '*ill' *women should attend ANC. In other words, women do not see the need to seek care during pregnancy except when they are unwell or to get a card. This is contrary to the view of some medical professionals who perceive that every pregnancy requires medical care and hence women must attend ANC in order to have safe deliveries. Yet it is generally known that pregnancy and childbirth are biological and physiological events which are embedded in a social and cultural setting [29]. Therefore, for ANC to be effective, it needs to be perceived from both medical and social/cultural perspectives [29]. In addition, there is a need to redefine ANC from "drinking medicine" to caring for a healthy pregnancy, while keeping in mind that comprehension and interpretation of health messages are different and new information interacts with previous ideas into "syncretic models" [30].

Other important barriers to seeking care that need to be addressed include transport and reports of requests for illegal payment of health workers by mothers. In addition, men will need to be targeted as key allies in improving ANC attendance. Current strategies in Uganda tend to focus mainly on women, yet it is men who provide financial support and also make decisions to seek care.

Our findings on ANC are supported by a recent systematic review of factors affecting ANC in low-income countries by Simkhada et.al. which found that the key factors affecting antenatal care uptake include: cultural beliefs and ideas about pregnancy, maternal and husband's education, marital status, availability and cost of ANC, household income, women's employment, media exposure and having a history of obstetric complications [31].

We believe that a community-based approach to dialogue with parents and communities to increase "demand" for ANC [32] coupled with a health system intervention to improve the "supply" of quality, affordable ANC will improve ANC attendance in Uganda, as it did in Nepal where women's participatory groups helped reduce neonatal mortality, increased supervised deliveries and ANC attendance [12].

### Birth preparedness and supervised delivery

We found that most women prefer to have a supervised delivery, but in practice often do not manage, mainly because having a supervised delivery is too costly for them to afford. Although user-fees were officially abolished in Uganda since 2001, in practice couples need to incur costs for drugs and other medical supplies. These high costs incurred by couples may be one of the major explanations as to why supervised deliveries in Uganda have remained low for over a decade despite high ANC attendance rates and improved physical access to health facilities. In Uganda, 92% of pregnant women attend ANC at least once, and 72% of the population now lives within a distance of 5 km from the nearest health facility [19].

Based on these findings, we suggest that the issue of BP needs to be revisited with the aim of reducing the financial burden on couples [33]. In addition, mothers told us that most labour pains start at night when there is no money and adequate transport to get to health facilities which provide delivery care, implying the need to consider locally available transport as well as waiting shelters for pregnant women in under-served areas [33-37] if supervised delivery care is to be increased.

### Postnatal care for the mother and baby

Our findings show that postnatal care was barely present in practice and was not promoted by service providers or through the community outreach activities. However, most recommended care practices for the newborn were said to be acceptable to both health providers and community members. Notable exceptions were delayed bathing and putting nothing on the umbilical cord, which were relatively unacceptable. Early bathing within the first 8–12 hours is common and a range of different substances are put on the cord.

Therefore, in order for effective newborn interventions to take effect, the acceptable recommended practices will need strengthening. However, special efforts including compromise practices will need to be defined and promoted for those recommended practices which are not easily accepted. Thus, in order to keep babies warm at birth, the recommended practice of delayed bathing should be communicated better, or a compromise of wiping the baby with a clean warm piece of cloth will need to be promoted along with skin-to-skin care. Similarly, there is need to define some harmless practices for cord-care. We find that this is one of the key areas where a community based approach has a major contribution since most births occur outside health units and facility attendance is not deemed feasible in the first week after birth due to cultural, transport and financial reasons.

### Methodological considerations

While we selected mainly respondents who had recently given birth, efforts were made to encourage them to give their actual experiences, for some recommended practices respondents probably gave us general attitudes prevalent in their communities. Another possible limitation is that the study focused on neonatal care practices and did not target maternal issues other than when they were thought to impact on the newborn. While this was a study in one region only, we believe that some of our findings are generalizable to other parts of Uganda. However, we believe the methodology used to elicit the findings can and should be used across regions and countries in the formative stages of intervention development.

## Conclusion

We found that, except for a few, the internationally recommended evidence-based practices are acceptable to the community and to health service providers, but that they are not practiced due to both health systems and community barriers. Women attend ANC mostly when they feel ill or in order to get an ANC card, suggesting that communities seem to perceive pregnancy and childbirth differently from the way health professionals do. Postnatal care is non-existent in this region.

Communities should be educated on the realistic expectations of ANC and on the fact that pregnancy is not an illness but has risks which may need medical help. The financial burden on families for birth preparedness must be reduced if skilled attendance at delivery is to increase, which requires better supply availability in the health facilities. Male involvement is essential and promotion of waiting shelters at selected health units should be considered in order to increase access to supervised deliveries. Initiatives to scale-up the recommended practices for improving neonatal health in Sub-Saharan Africa should follow rapid appraisal and adaptation of packages to address the local health system and socio-cultural situation. This will inform message development for behavior change communication and allow prioritization of recommended practices which have the greatest ease of introduction and likely high impact on reducing newborn mortality in the local situation.

## Competing interests

The authors declare that they have no competing interests.

## Authors' contributions

PW, GWP and SP conceived the study. PW, JK, SN, GWP and SP took part in designing the study, in tools development, analysis and manuscript writing. PW and MK developed tools, did field work, analysed the material and drafted the manuscript. All authors approved the final manuscript.

## Pre-publication history

The pre-publication history for this paper can be accessed here:


